# Supplementary material for: Genome-wide association, prediction and heritability in bacteria with application to Streptococcus pneumoniae
Source: NAR Genom Bioinform. 2022 Feb 22;4(1):lqac011. doi: 10.1093/nargab/lqac011 (PMC8862724; doi:10.1093/nargab/lqac011)
Supplement: lqac011_Supplemental_File [file lqac011_supplemental_file.pdf]

Genome-wide association, prediction and heritability in bacteria - Supplementary Information

Sudaraka Mallawaarachchi[1,*], Gerry Tonkin-Hill[2], Nicholas J. Croucher[3], Paul Turner[4,5], Doug Speed[6,7,8], Jukka Corander[2,9,10], David Balding[1,8,11,*]

1 Melbourne Integrative Genomics, School of Mathematics and Statistics, University of Melbourne, VIC, Australia
2 Parasites and Microbes, Wellcome Sanger Institute, Cambridge, UK
3 Faculty of Medicine, School of Public Health, Imperial College, London, UK
4 Cambodia-Oxford Medical Research Unit, Angkor Hospital for Children, Siem Reap, Cambodia
5 Centre for Tropical Medicine and Global Health, Nuffield Department of Medicine, University of Oxford, Oxford, UK
6 Aarhus Institute of Advanced Studies (AIAS), Aarhus University, Denmark
7 Bioinformatics Research Centre, Aarhus University, Denmark
8 UCL Genetics Institute, University College London, United Kingdom
9 Department of Biostatistics, Faculty of Medicine, University of Oslo, Oslo, Norway.
10 Helsinki Institute of Information Technology, Department of Mathematics and Statistics, University of Helsinki, Helsinki, Finland.
11 School of BioSciences, University of Melbourne, VIC, Australia
* (sudaraka.mallawaarachchi,dbalding)@unimelb.edu.au

S1 Appendix. Results from the carriage duration analysis using the dataset comprising all 1 612 isolates sampled during a positive episode

We performed association testing on the dataset comprising all isolates (1 612) that were linked to a carriage episode. An additional 733 accessory genes were tested here, none of which showed association, and Gap and SNP tests were performed on 77 614 and 115 745 core genome variants in total, respectively, with 44 119 subject to both tests. Four sites (gap tested only) were significant ($p = 4 \times 10^{-9}$) at a Bonferroni corrected threshold of 0.01. These all correspond to an insertion represented in 15 sequences, at basepair positions 1 522 542, 1 522 896, 1 522 934 and 1 522 964. This region is within the MM1-type prophage of the ATCC 700669 genome. An association with CD was previously reported at a nearby locus using k-mer analysis [1]. The 15 insertion sequences have a mean CD of 308 days (SD 183), compared with 145 (SD 125) for the 1 597 gap sequences. However, the association signal largely comes from 6 isolates from the same 517-day CD episode and requires further investigation to confirm its significance. Estimates of h^2 (Table 1) were naturally higher for this larger dataset where 337 episodes are over-represented by isolates.

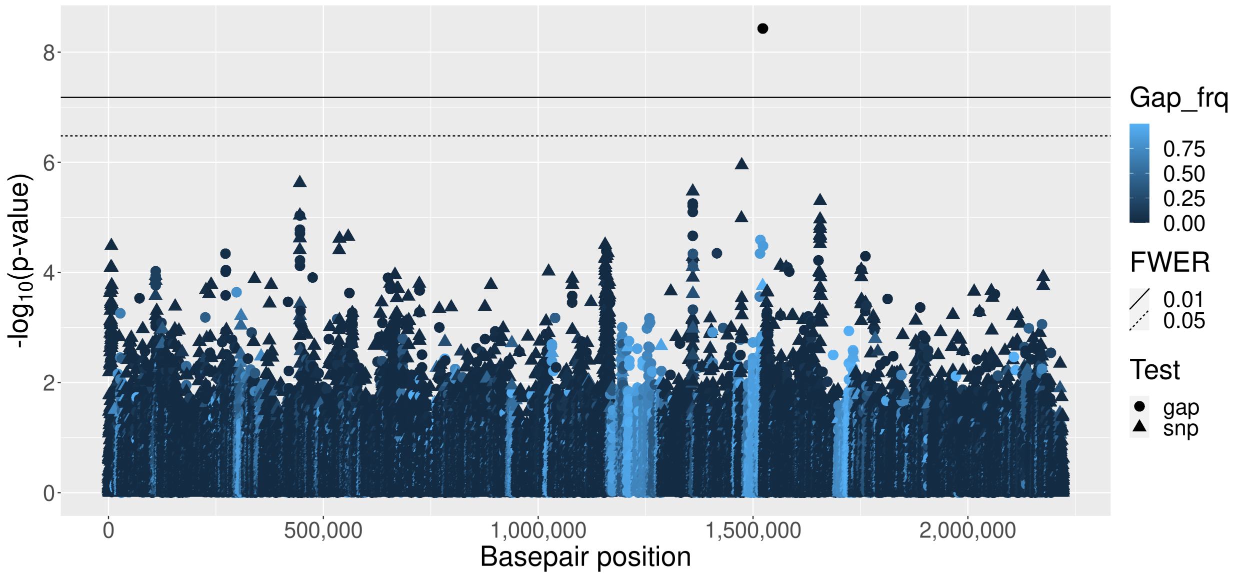

Figure 1: **Carriage duration (CD) Manhattan plot for core genome variants.** Accessory genes are not shown. See legend for shading that indicates gap frequency and symbol shape indicating whether the test of association was based on gaps or SNP variation. Basepair positions are obtained from the ATCC700669 reference genome alignment.

Table 1: **Heritability estimates ($\hat{h}^2$).** Top and bottoms rows show $\hat{h}^2$ for the core and pan genomes, respectively.

Phenotype (log)	Pyseer LMM	Pyseer wg-enet	LDSC
CD - all isolates	0.61	0.52	0.52
with Accessory genes	0.61	0.58	0.54

S2 Appendix. Phenotype prediction using major allele frequency coded variants

Table 2: **Phenotype prediction with major allele coded variants.** Mean squared error (MSE) and the correlation between observed and predicted test values using 10-fold (10F) and leave-one-strain-out (LOSO) cross validation (CV). Predictions were performed using a wg-enet model in glmnet. Approximately 1.5% of available predictors were used for CD and 1% were used for the two MIC phenotypes.

Phenotype	10F CV		LOSO CV	
(log scale)	MSE (SE)	Cor (SE)	MSE (SE)	Cor (SE)
CD	0.100 (0.005)	0.540 (0.022)	0.113 (0.004)	0.457 (0.024)
Ceftriaxone MIC	0.031 (0.002)	0.910 (0.005)	0.086 (0.004)	0.744 (0.012)
Penicillin MIC	0.042 (0.003)	0.909 (04005)	0.120 (0.005)	0.731 (0.013)

S3 Appendix. Genes identified by pyseer-LMM and treeWAS analyses

The LMM MA test identified 817 core-variant associations for ceftriaxone MIC, 524 of which were in 22 genes, 13 of them also identified by the gap/SNP test. For penicillin MIC, 602 associations were identified, 444 of which were mapped to 16 genes, also 13 in common with the gap/SNP test. No accessory gene associations were identified for either MIC phenotype.

Table 3: **Associated genes identified from pyseer-LMM.** All variants above the significance threshold in any test is checked for annotations in the ATCC700669 reference genome for associated genes.

Phenotype (log)	Core genes	Accessory genes
CD - 1-isolate/episode	-	-
CD - all isolates	-	-
Ceftriaxone MIC	pbpX, pbp1A, aliA, mraY, recU, mraW, gnd, clpL, penA, csrR, dexB, luxS, accD, aldB, comFC, folC, lepA, mtsC, pabB, rplK, valS, wzh	-
Penicillin MIC	pbp1A, aliA, pbpX, recU, mraY, wzg, dexB, gnd, luxS, clpL, csrR, leuB, leuS, potD, recO, smc	-

TreeWAS only identified 140 and 66 core-genome associations for ceftriaxone and penicillin MIC, respectively.

Table 4: **Associated genes identified from treeWAS.** All variants above the significance threshold in any test is checked for annotations in the ATCC700669 reference genome for associated genes.

Phenotype (log)	Core genes	Accessory genes
CD - 1-isolate/episode	purF, polA	-
CD - all isolates	dgk, lacF, msrAB, murM, lacG2, alsS, polC, hrcA, dpr	-
Ceftriaxone MIC	pbp1a, pbp2b, pbp2x, mraY, clpL, recU, aliA, dexB, gnd	-
Penicillin MIC	pbp1a, pbp2b, pbp2x, recU, mraY, clpL, luxS, gnd	-

Supplementary Figures

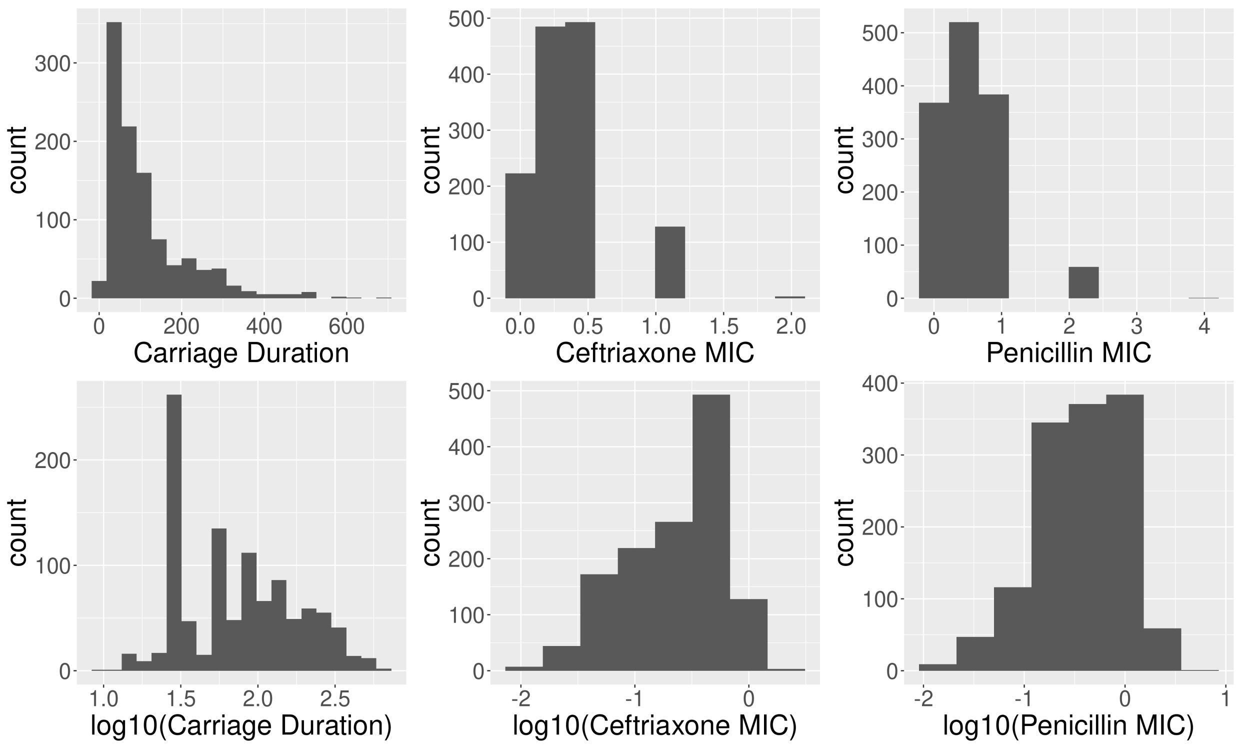

Figure S 1: **Phenotype distribution.** Top and bottom rows show the distribution of the three phenotypes before and after $\log_{10}$ transformation.

References

[1] Lees, J. A., Croucher, N. J., Goldblatt, D., Nosten, F., Parkhill, J., Turner, C., Turner, P., and Bentley, S. D. (2017) Genome-wide identification of lineage and locus specific variation associated with pneumococcal carriage duration. *Elife*, **6**, e26255.

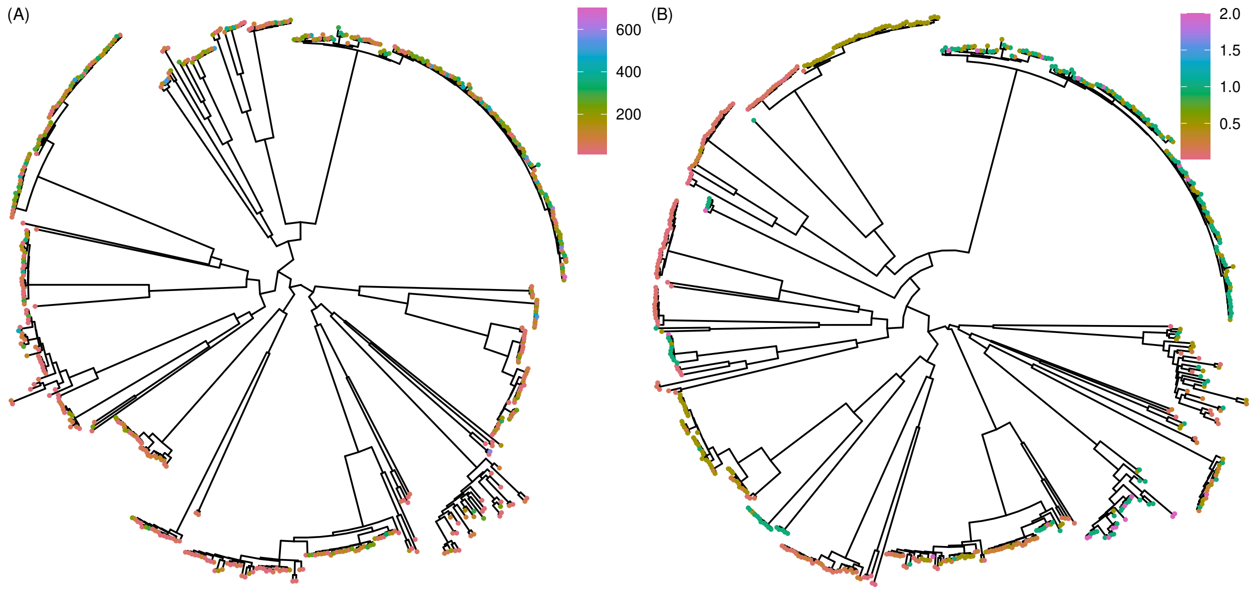

Figure S 2: **Phylogenetic trees from the ClonalFrameML analysis.** Mid-point rooted, 'recombination-aware' tree structure for (A) 1 047 isolates with carriage duration phenotype (measured in days and indicated by tip colour) and (B) 1 332 isolates with MIC phenotype (measured in µg ml^{-1} and tip colour indicates the distribution of penicillin MIC)

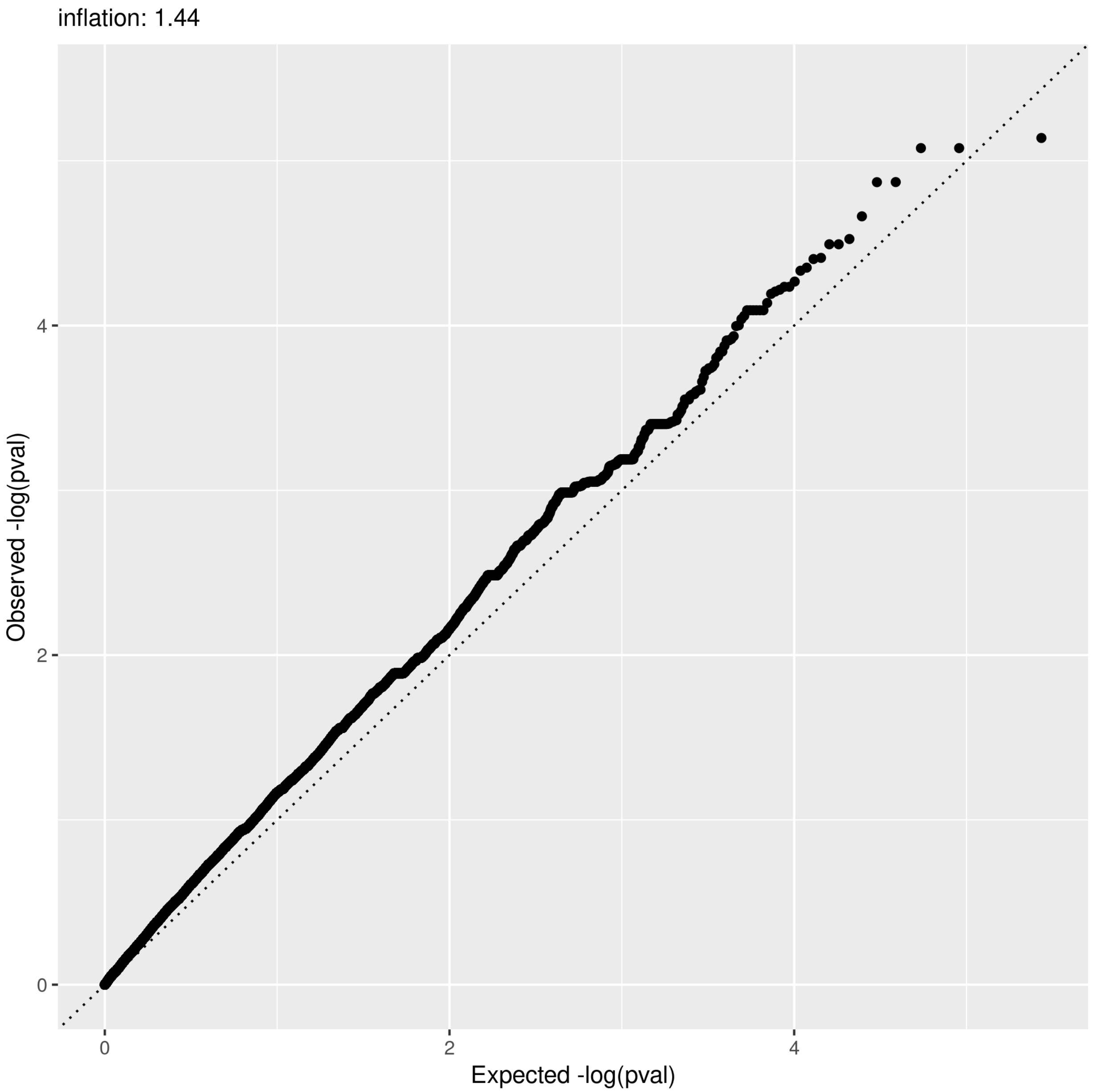

Figure S 3: **QQ plot for carriage duration from the GAP/SNP analysis.**

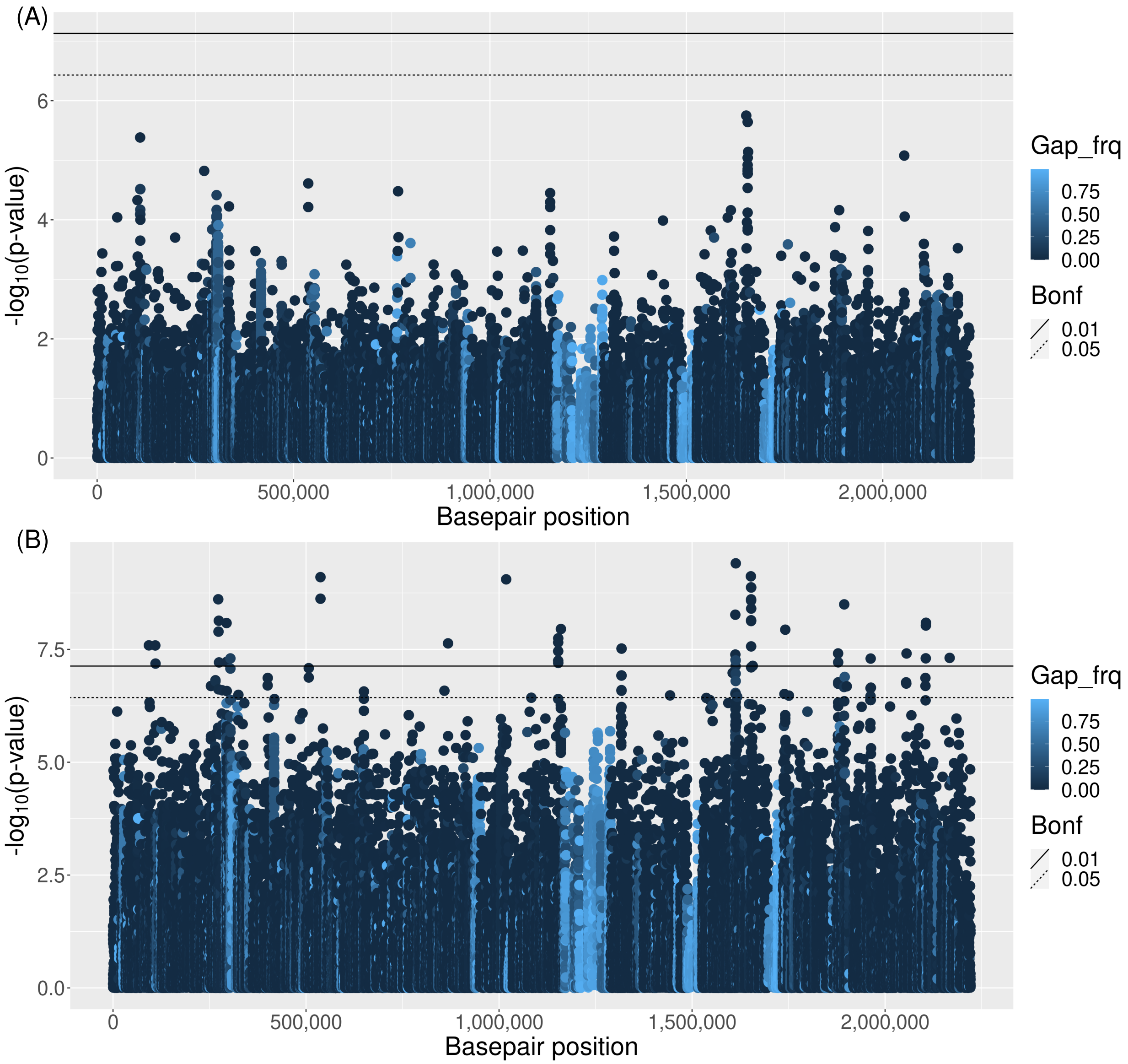

Figure S 4: **Manhattan plot from MA tests of association with CD.** Testing was performed with (A) LMM and (B) FEM models. LMM did not identify any significant associations, whereas FEM identified 92 associations with GIF = 2.53, indicating genome-wide inflation due to unsatisfactory control of population structure. In FEM, population structure correction was performed using FastBAPS cluster indicator covariates. Point colour indicates the gap frequency at each site and the horizontal lines indicate Bonferroni corrected significance thresholds.

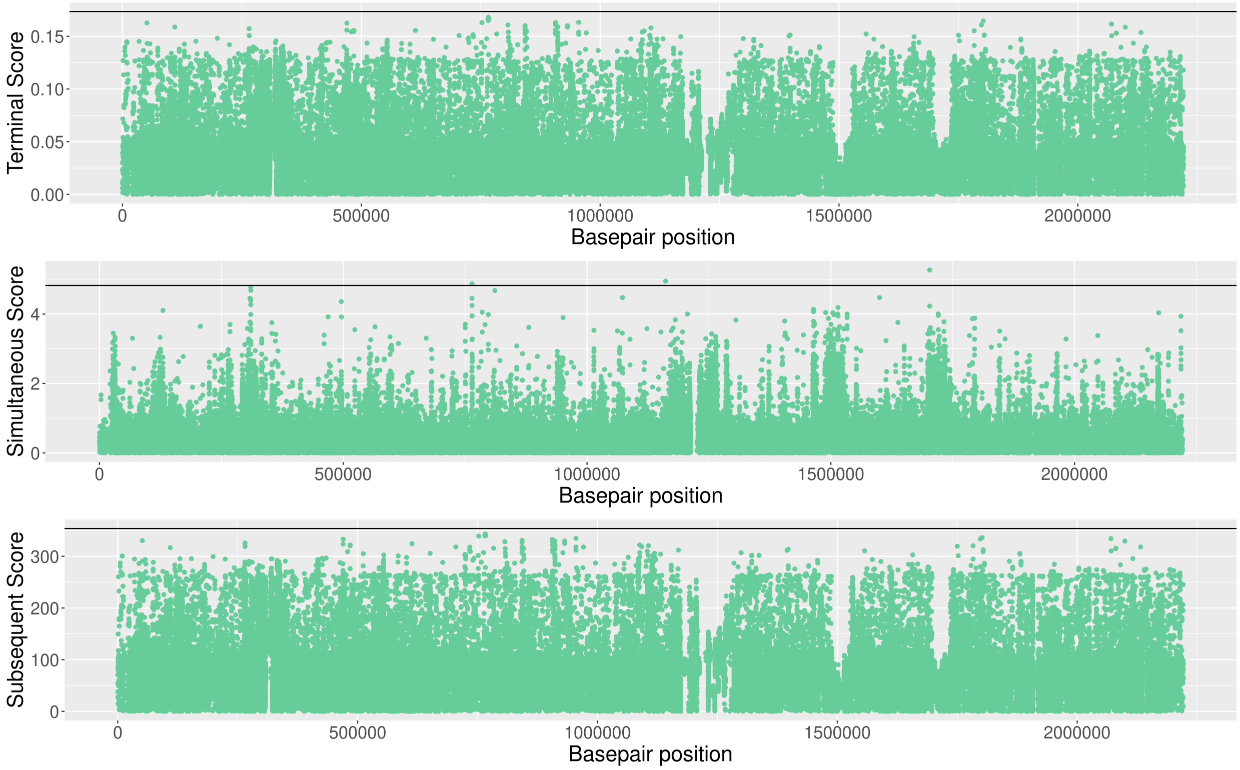

Figure S 5: **treeWAS analysis for CD.** Manhattan plots for (top) Terminal (middle) Simultaneous and (bottom) Subsequent scores are shown, where three hits are identified from the simultaneous test.

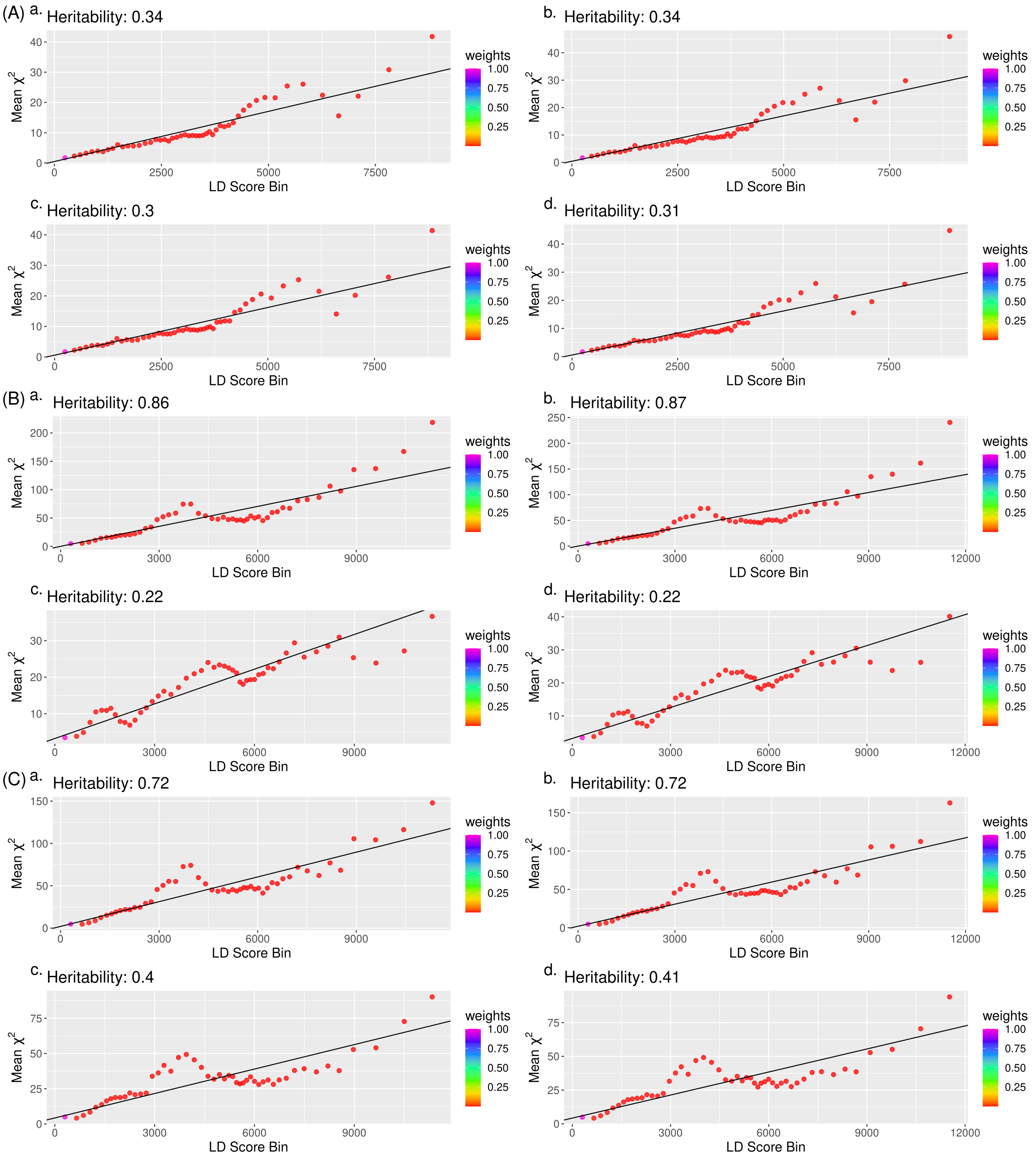

Figure S 6: **LDSC analyses for all phenotypes.** LDSC plots for (A) CD, (B) ceftriaxone MIC and (C) penicillin MIC. In each figure, subplots correspond to the **a.** core genome **b.** pangenome **c.** core genome w/o DR and **d.** pangenome w/o DR analyses and the $\hat{h}^2$ are reported in Table 2.

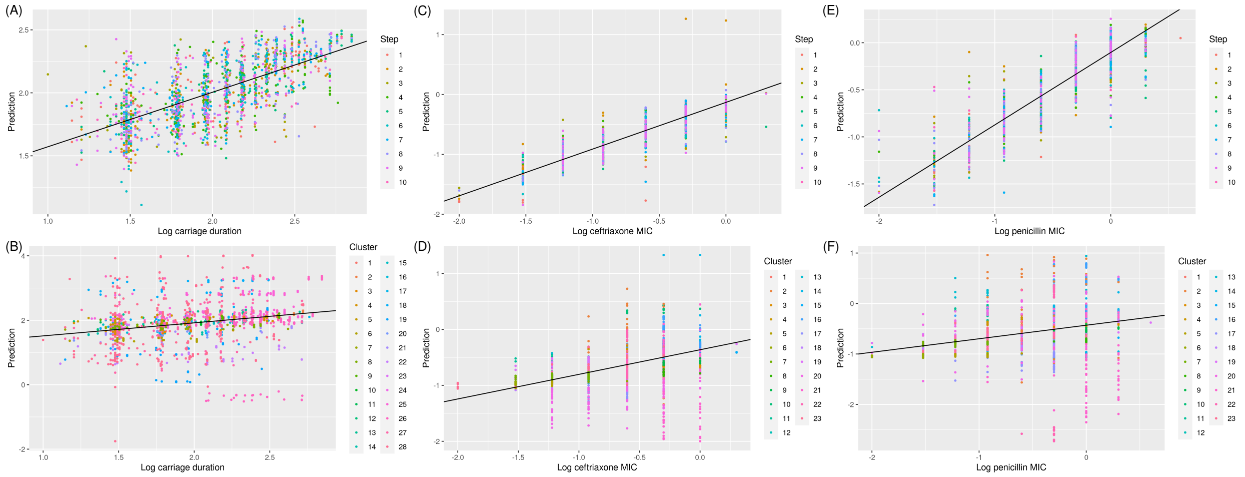

Figure S 7: **Prediction accuracy with MA and frequency coding.** Allele frequency coding generally increases the correlation and reduces the mean squared error of prediction for all three phenotypes across folds and clusters. Note that the Mean squared error and correlation values here are averaged across folds and clusters, and are different from the overall accuracy results in Table Table 1 and S2 Appendix

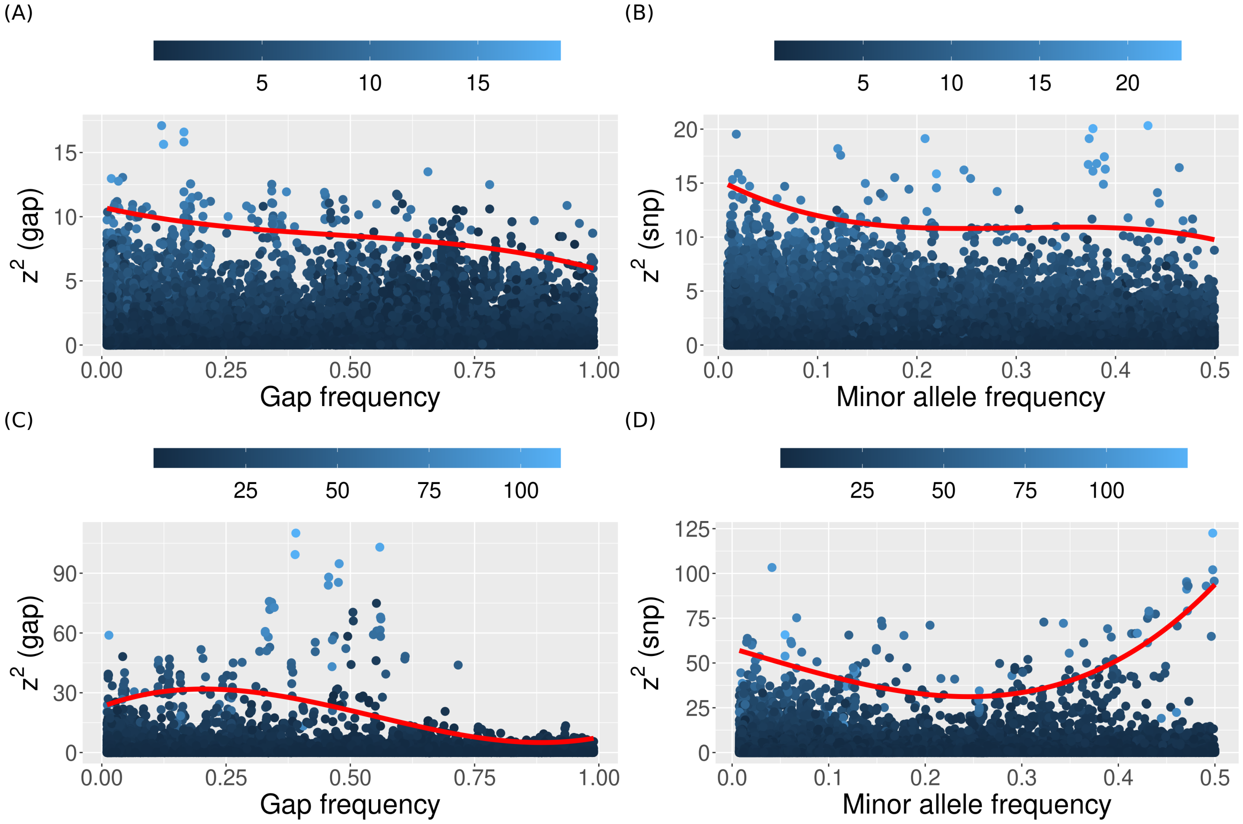

Figure S 8: **Variation in z^2 statistics with variant frequency** Each point shows the z^2 statistic of a (A, C) gap and (B, D) SNP tested core genome variants for (A, B) carriage duration and (C, D) penicillin MIC. The x-axis respectively shows the gap and minor allele frequency for gap and SNP tested variants. Points are shaded according to the z^2 statistic from the MA test and the red curve shows the 4th order regression fit for the 90th percentile of data.

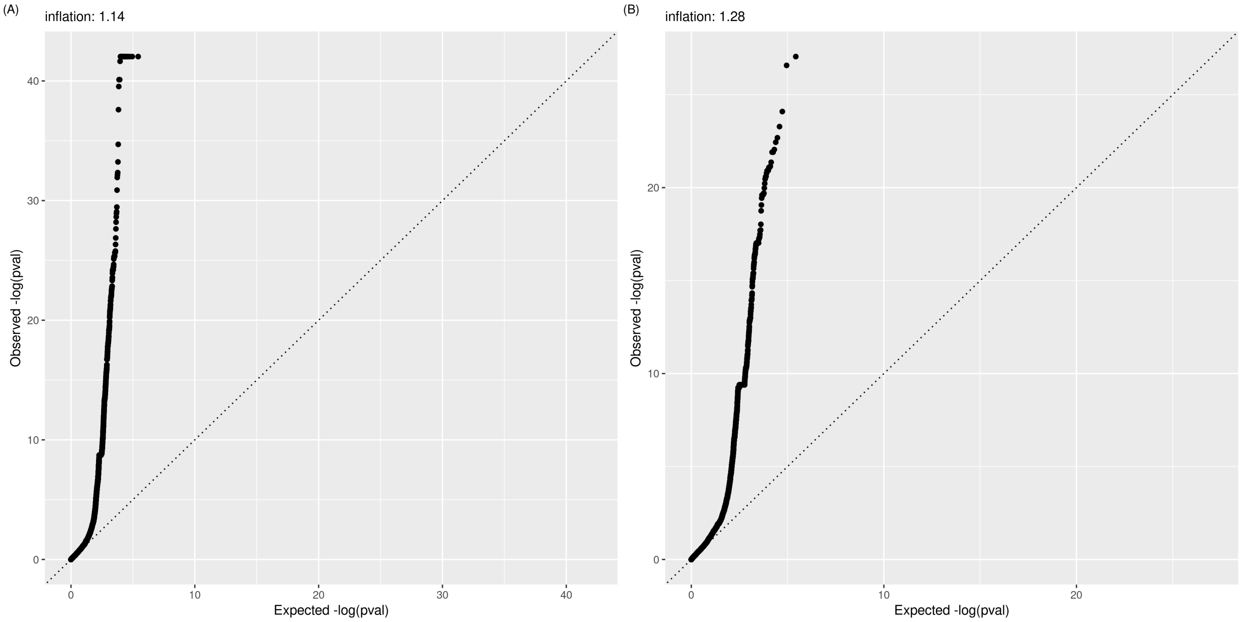

Figure S 9: **QQ plots for MIC phenotypes from the GAP/SNP analysis.** (A) ceftriaxone MIC (B) penicillin MIC.

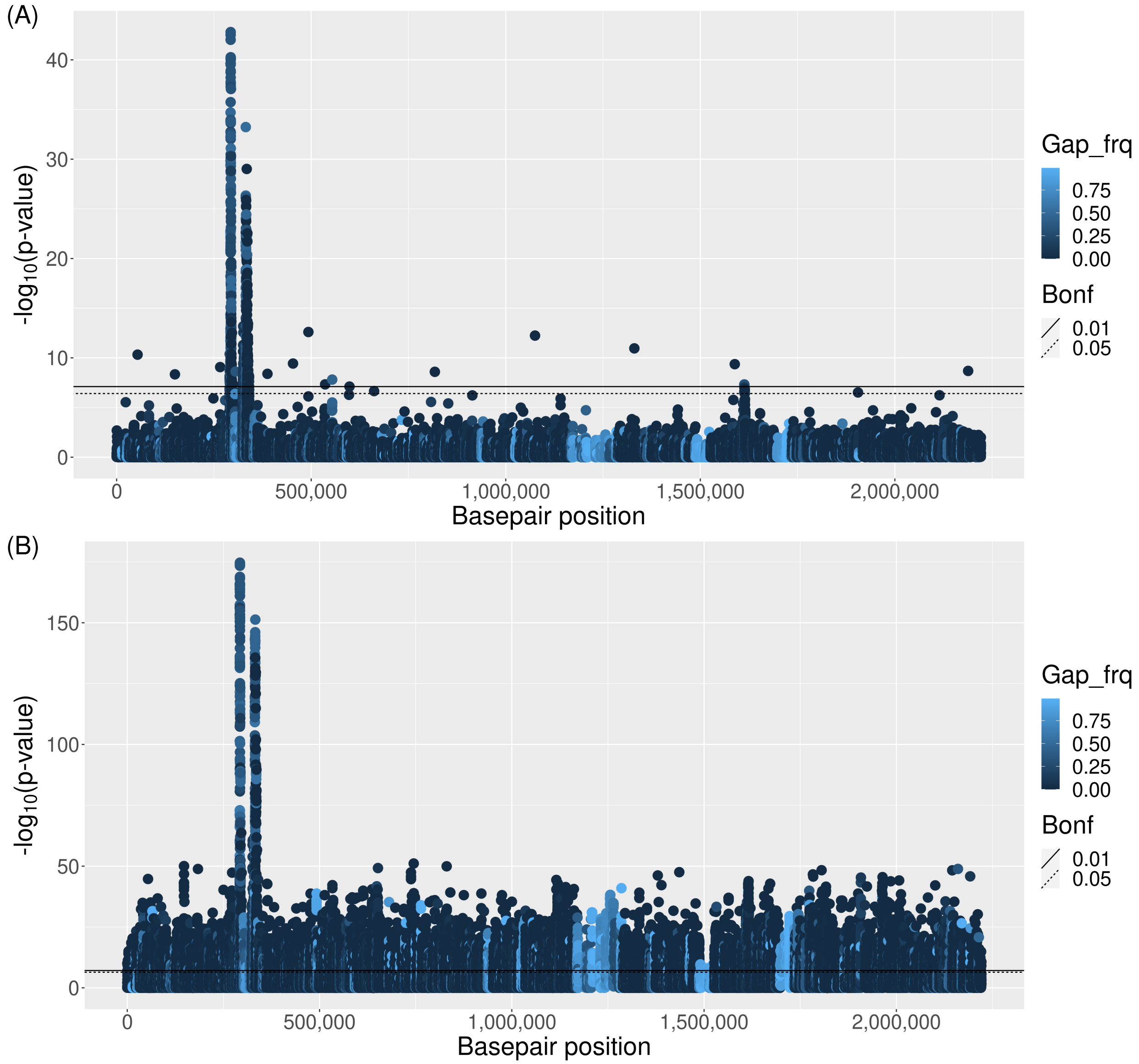

Figure S 10: **Major-allele test for ceftriaxone MIC.** Testing was performed using (A) LMM and (B) FEM models. FEM analysis identified 13 212 hits with GIF = 16.4. See caption in **??** for additional analysis and figure legend details.

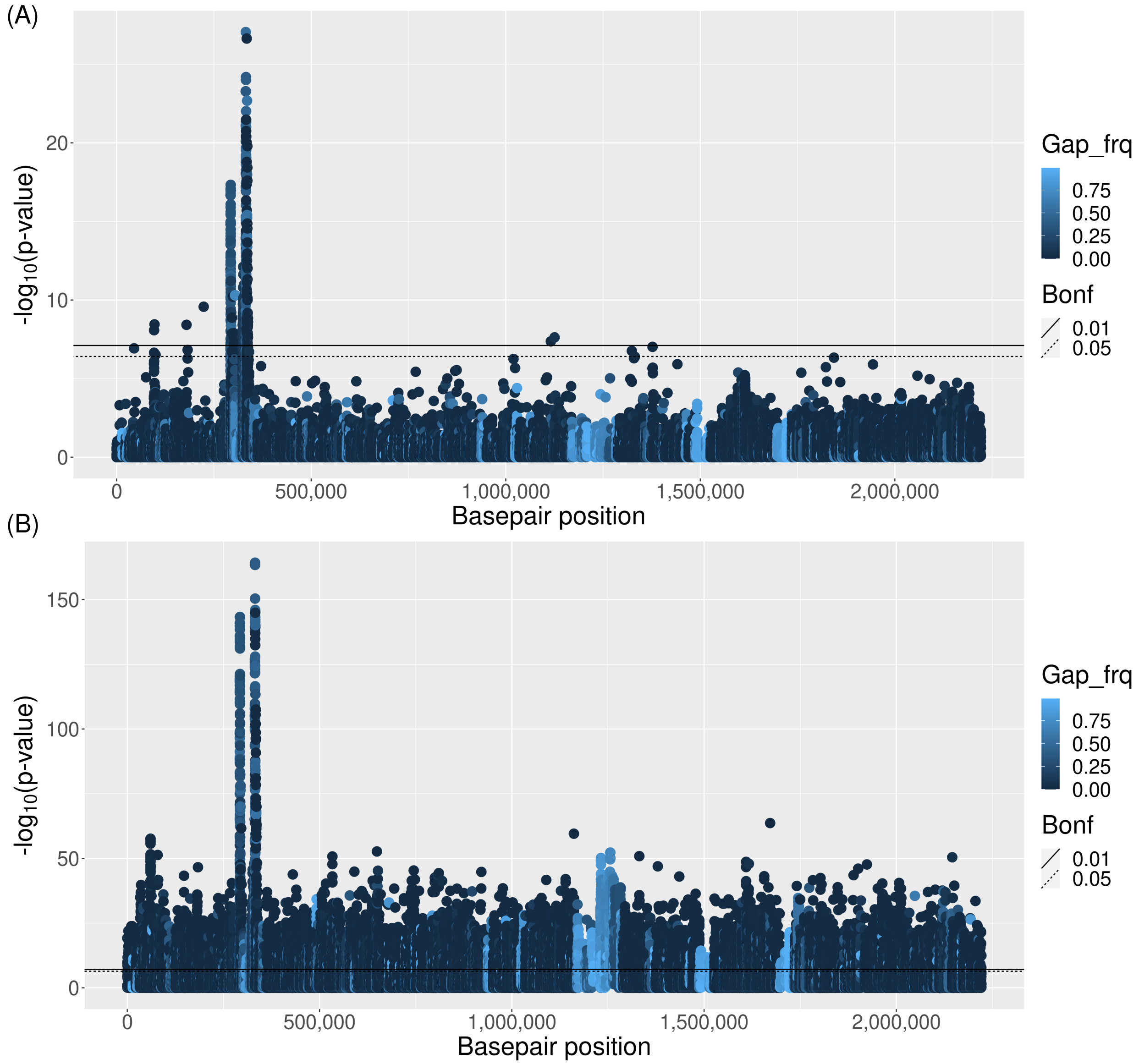

Figure S 11: **Major-allele test for penicillin MIC.** Testing was performed using (A) LMM and (B) FEM models. FEM analysis identified 23 636 hits with GIF = 17.0. See caption in **??** for additional analysis and figure legend details.

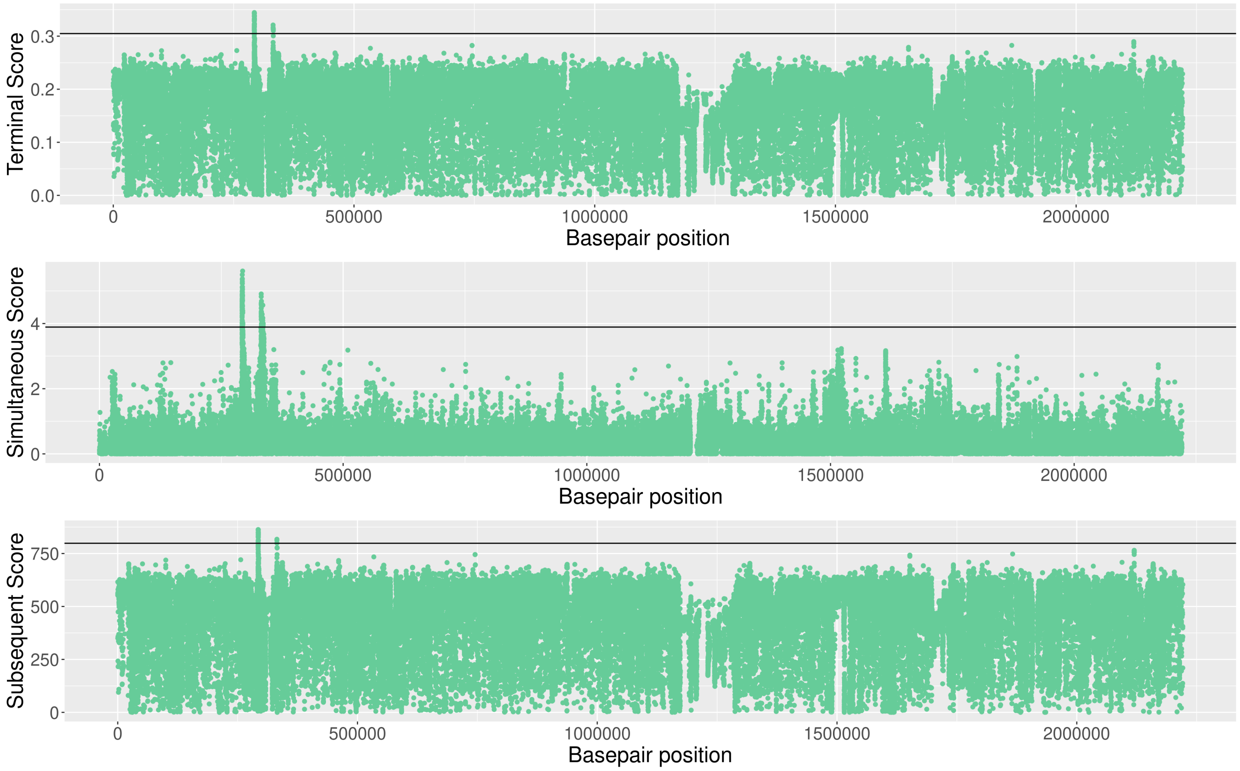

Figure S 12: **treeWAS analysis for ceftriaxone MIC.** Manhattan plots for (top) Terminal (middle) Simultaneous and (bottom) Subsequent scores.

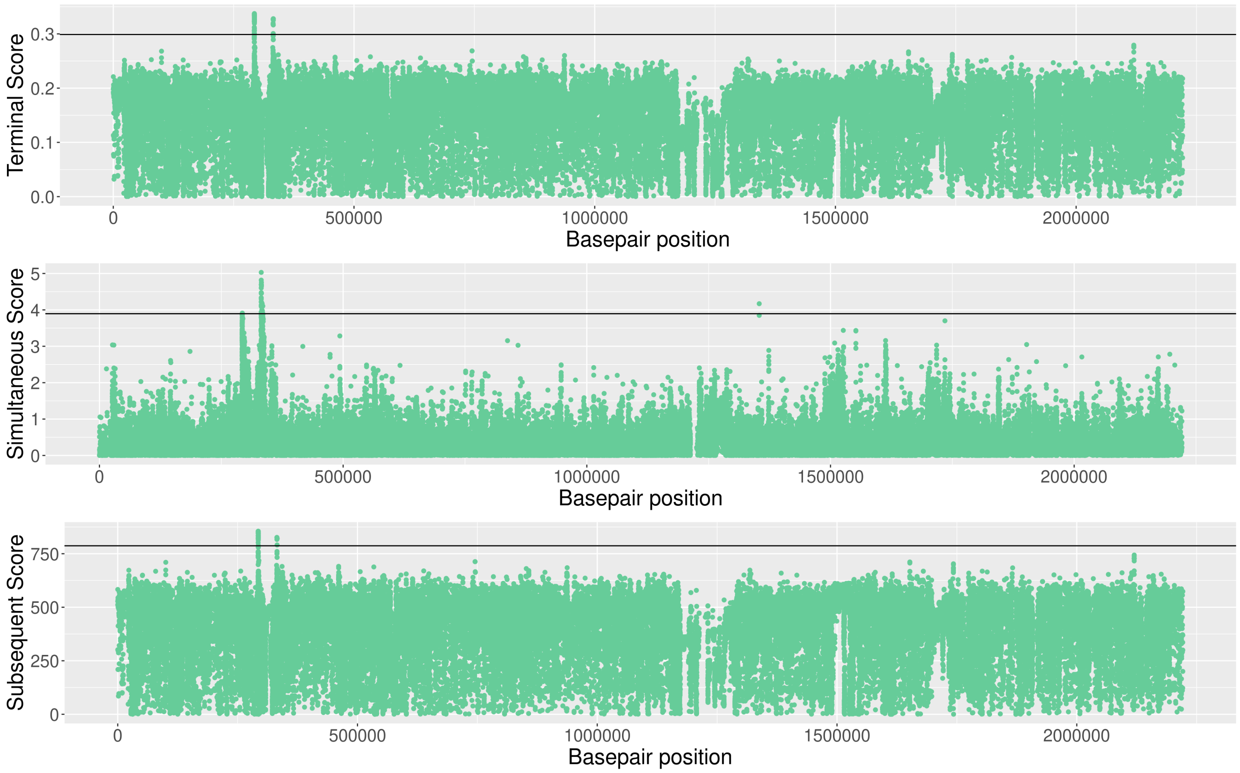

Figure S 13: **treeWAS analysis for penicillin MIC.** Manhattan plots for (top) Terminal (middle) Simultaneous and (bottom) Subsequent scores.

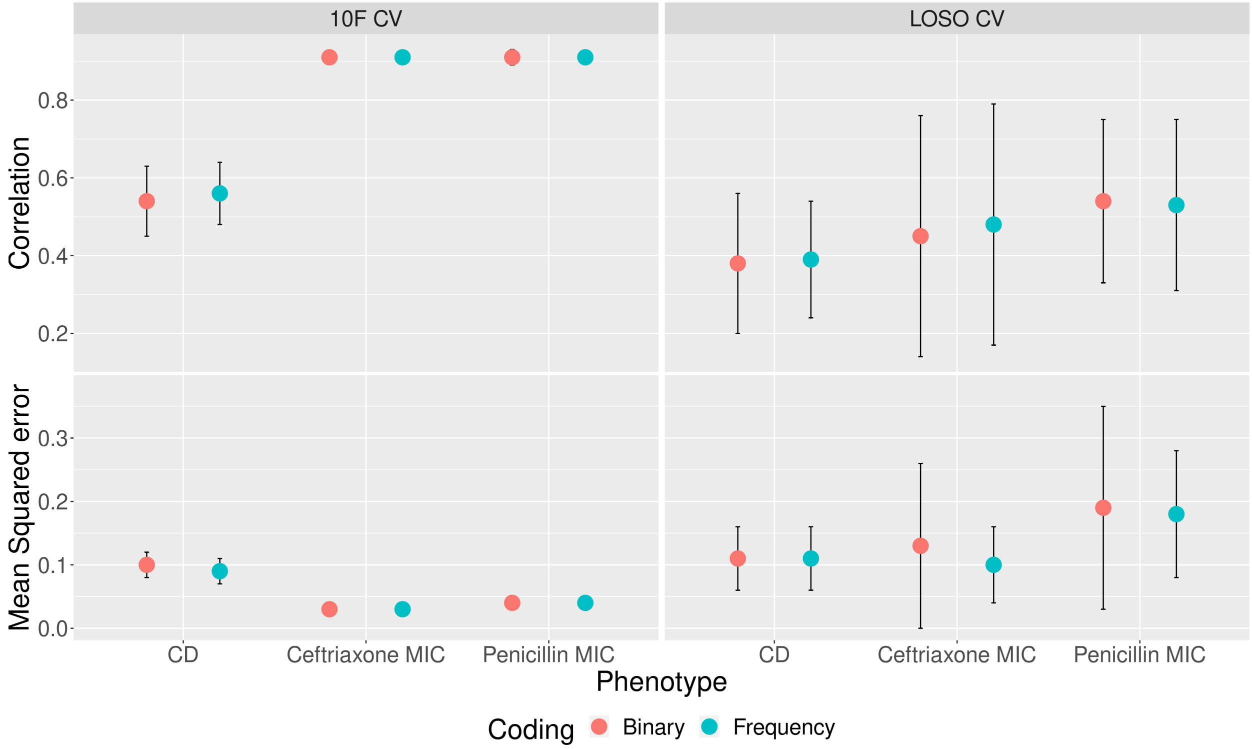

Figure S 14: **Prediction performance.** Prediction performance of (A,B) carriage duration, (C,D) ceftriaxone MIC and (E,F) penicillin MIC phenotypes, assessed using (A,C,E) 10F and (B,D,F) LOSO CV. The x and y axes denote the true and predicted values, respectively and point colour represents the fold or FastBAPS cluster. Mean squared error and correlation values in Table 1 and S2 Appendix are computed using all values shown here.
